# Protein-Pacing and Multi-Component Exercise Training Improves Physical Performance Outcomes in Exercise-Trained Women: The PRISE 3 Study †

**DOI:** 10.3390/nu8060332

**Published:** 2016-06-01

**Authors:** Paul J. Arciero, Stephen J. Ives, Chelsea Norton, Daniela Escudero, Olivia Minicucci, Gabe O’Brien, Maia Paul, Michael J. Ormsbee, Vincent Miller, Caitlin Sheridan, Feng He

**Affiliations:** 1 Human Nutrition and Metabolism Laboratory, Department of Health and Exercise Sciences, Skidmore College, Saratoga Springs, NY 12866, USA; sives@skidmore.edu (S.J.I.); chelseanorton1@gmail.com (C.N.); descuder@skidmore.edu (D.E.); ominicucci1@gmail.com (O.M.); gobrien@skidmore.edu (G.O.); maiapaul@yahoo.com (M.P.); vin.miller@gmail.com (V.M.); csherida@skidmore.edu (C.S.); fhe@csuchico.edu (F.H.); 2 Institute of Sports Sciences & Medicine, Department of Nutrition, Food and Exercise Sciences, Florida State University, Tallahassee, FL 32306, USA; mormsbee@fsu.edu; 3 Department of Kinesiology, California State University, Chico, CA 95929, USA

**Keywords:** protein-pacing, exercise-trained women, PRISE, muscular fitness, augmentation index

## Abstract

The beneficial cardiometabolic and body composition effects of combined protein-pacing (P; 5–6 meals/day at 2.0 g/kg BW/day) and multi-mode exercise (resistance, interval, stretching, endurance; RISE) training (PRISE) in obese adults has previously been established. The current study examines PRISE on physical performance (endurance, strength and power) outcomes in healthy, physically active women. Thirty exercise-trained women (>4 days exercise/week) were randomized to either PRISE (*n* = 15) or a control (CON, 5–6 meals/day at 1.0 g/kg BW/day; *n* = 15) for 12 weeks. Muscular strength (1-RM bench press, 1-RM BP) endurance (sit-ups, SUs; push-ups, PUs), power (bench throws, BTs), blood pressure (BP), augmentation index, (AIx), and abdominal fat mass were assessed at Weeks 0 (pre) and 13 (post). At baseline, no differences existed between groups. Following the 12-week intervention, PRISE had greater gains (*p* < 0.05) in SUs, PUs (6 ± 7 *vs.* 10 ± 7, 40%; 8 ± 13 *vs.* 14 ± 12, 43% ∆reps, respectively), BTs (11 ± 35 *vs.* 44 ± 34, 75% ∆watts), AIx (1 ± 9 *vs.* −5 ± 11, 120%), and DBP (−5 ± 9 *vs.* −11 ± 11, 55% ∆mmHg). These findings suggest that combined protein-pacing (P; 5–6 meals/day at 2.0 g/kg BW/day) diet and multi-component exercise (RISE) training (PRISE) enhances muscular endurance, strength, power, and cardiovascular health in exercise-trained, active women.

## 1. Introduction

There continues to be a heightened interest in healthy lifestyles among women. However, limited data is available on combined nutrition and exercise training interventions that quantify changes in fitness-related outcomes such as muscular strength, power and endurance, aerobic fitness, flexibility, and balance in this population. Recently, we demonstrated that a protein-pacing diet alone (P; 5–6 meals/day at >1.4 g/kg BW protein/day; 20–25 g protein/meal) [1] combined with a multi-mode (RISE; resistance, interval, stretching, endurance) exercise training intervention (PRISE) results in greater reductions in total and regional (abdominal/visceral) fat mass, greater gains in lean mass, and enhanced cardiometabolic health compared to a combined protein-pacing (P) and traditional resistance training intervention in obese/overweight women [2]. Thus, it is of interest to examine the efficacy of the PRISE lifestyle (nutrition/exercise) program in improving physical performance and body composition outcomes in lean, fit women.

Given the paucity of scientific investigations examining fitness/performance outcomes in response to nutrition and exercise interventions in physically active women, the primary aim of the present study was to compare PRISE (5–6 meals/day at 2.0 g/kg BW/day) to normal protein intake and RISE training (CON) on fitness-related performance (strength, power, aerobic fitness, flexibility, balance), body composition, and cardiometabolic outcomes in healthy, exercise-trained women. We hypothesized, given our previous findings in obese adults [2], that PRISE would result in improved fitness-related performance, body composition, and cardiometabolic health outcomes when compared to CON.

## 2. Materials and Methods

### 2.1. Participants

**Figure 1:**
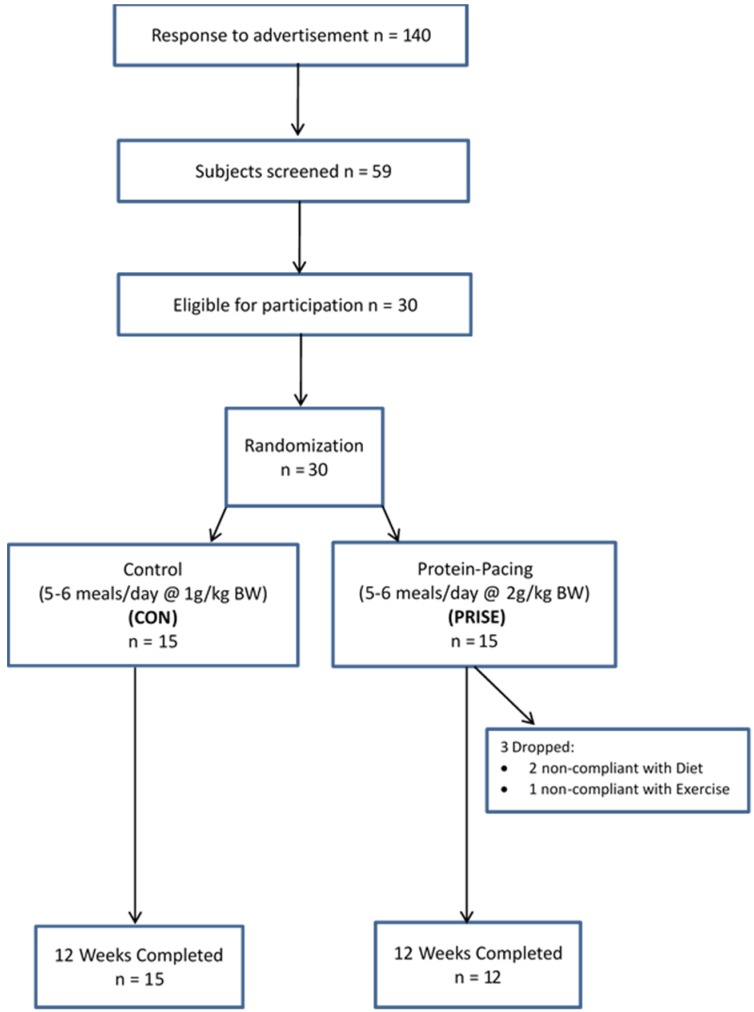
CONSORT flow chart of participants during the intervention.

A total of 140 women from the Saratoga Springs, NY area, responded to emails, flyers, and local newspapers to advertisements regarding the study. A total of 59 subjects were initially screened, of which 30 were eligible for participation (Figure 1).

Participants were nonsmoking, healthy, exercise-trained women with no known cardiovascular, renal, or metabolic diseases as assessed by a medical history and a comprehensive medical examination. All participants were highly active (minimum of >30 min, 4 day/week of structured physical activity), lean (BMI < 25 kg/m^2^; % body fat <30%), middle-aged (25–55 years), and weight stable (±2 kg) for at least 6 months prior to the beginning of the study assessed through questionnaire. All participants provided informed written consent prior to participation, and the study was approved by the Human Subjects Institutional Review Board of Skidmore College (IRB #: 1401-382). All experimental procedures were performed in accordance with the Federal Wide Assurance and related New York State regulations, which are consistent with the National Commission for the Protection of Human Subjects of Biomedical and Behavioral Research and in agreement with the Helsinki Declaration as revised in 1983. This study was registered with ClinicalTrials.gov Identifier: NCT02593656.

### 2.2. Experimental Design

#### Study Timeline

Participants were randomly assigned to one of two groups: (1) protein pacing and multi-mode exercise training (PRISE; *n* = 15; 5–6 meals/day at 2.0 g/kg BW/day) or (2) normal protein and multi-mode exercise training (CON; *n* = 15; 5–6 meals/day at 1.0 g/kg BW/day). This level of protein intake was used (≤2.0 g/kg BW per day) because it is regarded as safe and is not associated with any adverse effects on renal function (such as blood urea nitrogen, creatinine, glomerular filtration rate, and creatinine clearance). All participants performed the identical RISE exercise training program consisting of 4 days/week of closely supervised and monitored progressive exercise training for 12 weeks (Table S1). All testing procedures (see below) were administered pre-intervention (Week 0) and post-intervention (Week 13) unless otherwise noted. Upon arrival at the laboratory, anthropometric and body composition measurements and blood sampling for subsequent analysis were performed.

### 2.3. Nutrition Intervention

Meal plans were identically matched in terms of total kcals, meal frequency and timing, and dietary support. By design, the only difference between the two groups was the amount of protein (1.0 *vs.* 2.0 g/kg BW per day). Additional supplementation (daily multi-vitamin/minerals, and caffeine and electrolytes on workout days) was also provided to participants and differed only by the type of product manufacturer. Participants in both groups were provided detailed meal plans designed by a registered dietitian and instructed to follow the meal plans throughout the 12-week intervention (Table S2). The registered dietitian met with participants weekly for the first two weeks and thereafter on an “as needed” basis. In addition, investigators met with participants a minimum of four days per week to answer questions and reinforce meal plans. To facilitate adherence to the meal plans, food was provided to both groups.

PRISE meal plans included protein-pacing (P; 5–6 meals/day at 2.0 g/kg BW/day) on all days, three of which were whey protein-supplemented (IsaPro^®^: 150 kcals, 27 g protein, 3 g carbohydrate, 1.5 g fat; IsaLean Pro^®^: 280 kcals, 36 g protein, 21 g carbohydrate, 6 g fat; and IsaLean Bars^®^: 210 kcals, 18 g protein, 28 g carbohydrate, 5 g fat—Isagenix LLC, Chandler, AZ, USA). On exercise days, they were supplemented with a caffeine (e+^®^: 85 mg caffeine, 8 g carbohydrate) and electrolyte beverage (Replenish^®^: 35 kcals, 9 g carbohydrates, 110 mg sodium, 95 mg potassium—Isagenix LLC, Chandler, AZ, USA), and a multi-vitamin/mineral (Ageless Essentials^®^—Isagenix LLC, Chandler, AZ, USA) was taken every morning. It is important to note that the protein dosing was equivalent to >0.25 g/kg BW per meal, which has been shown to be the optimal intake for muscle protein synthesis [3]. Recently, it has been shown that women supplementing with whey protein and exercise training have increased lean mass compared to placebo supplements [4,5].

CON participants followed a similar healthy meal plan as PRISE but included a normal protein intake (5–6 meals/day at 1.0 g/kg BW/day on all days), three of which were supplemented (Nature Valley Protein Chewy Bars^®^: 190 kcals, 10 g protein, 14 g carbohydrate, 12 g fat; Nature Valley Sweet and Salty Nut Granola Bars^®^: 170, 4 g protein, 20 g carbohydrate, 8 g fat—General Mills, Inc., Minneapolis, MN, USA—and Horizon Organic Milk^®^: 150 kcals, 8 g protein, 22 g carbohydrate, 2.5 g fat—WhiteWave Foods Company, Inc. Broomfield, CO, USA). On exercise days, they also consumed a caffeine (tea or coffee with sweetener: ~85 mg caffeine, 8 g carbohydrate) and electrolyte beverage (Gatorade G2^®^: 45 kcals, 12 g carbohydrates, 250 mg sodium, 75 mg potassium—PepsiCo, Purchase, NY, USA) along with a multi-vitamin/mineral (One-A-Day Multivitamins^®^—Bayer, Whippany, NJ, USA) taken every morning. CON participants were also asked to return empty food packets to monitor compliance. It is important to note, by study design, the only macronutrient that was intentionally different between groups was the protein per kg BW. Participants in both groups were given a 1-week supply of the supplements and asked to return empty packets before they received the next week’s supply as a means of assessing their compliance. Both groups were provided equivalent nutritional support and similar caloric intakes throughout the 12-week intervention.

The timing of meals was an important component of the current study, and both groups consumed meals using an identical meal pattern schedule. On resistance (R) and interval (I) exercise days (See below), participants consumed a small snack (~250 kcals) prior and, on stretching (S) and endurance (E) days, arrived fasted but well hydrated and were allowed to consume the electrolyte beverage as needed on all exercise days. Breakfast was consumed after the exercise, and remaining meals were consumed at 3-h intervals throughout the remainder of the day. On non-exercise days, participants consumed breakfast within an hour of waking in the morning and remaining meals at 3-h intervals thereafter (Table S2).

### 2.4. RISE Exercise Training Protocol

Subjects in both groups underwent the same closely supervised/monitored progressive multiple exercise training regimen as described previously [2]. Briefly, the training program consisted of four specific types of exercise training: (1) resistance exercise; (2) interval sprints; (3) stretching/yoga/Pilates; and (4) endurance exercise (RISE training) (Table S1). Subjects underwent four exercise sessions per week, and the sessions rotated through the four types of exercise such that each of the four exercises was performed 1 day per week. To familiarize participants with the individual exercises and to ensure compliance, all training sessions were performed in the Skidmore College Sports Center under the close supervision of the research team. Intensity level was monitored at every exercise session with heart rate monitors (Polar H7, Polar Electro, Lake Success, NY, USA) to ensure subject safety and proper compliance with the exercise program.

Specific details of the four types of exercises that comprise the RISE training have been previously published [2,6] and are shown in Table S1. Briefly, the resistance (R) training sessions were completed within 60 min and consisted of a dynamic warm-up, footwork and agility, lower and upper body resistance, and core exercises, all performed at a resistance to induce muscular fatigue in 10–15 repetitions and for 2–3 sets. A 30-s recovery was provided between sets, and a 60-s recovery was allowed between different exercises. The sprint interval (I) training sessions were completed within 35 min and consisted of either 7 sets of 30 s “all-out” with a 4-min recovery or 10 sets of 60 s “almost all-out” with 2 min of rest after each interval. Participants were allowed to perform the sprints using any mode of exercise. The stretching (S) routine incorporated traditional yoga poses with additional stretches and Pilates movements, providing a total body stretching, flexibility, and strengthening workout. All sessions were completed within 60 min and were led by a certified yoga instructor. Finally, endurance (E) exercise training was performed for 60 min at a moderate pace (60% of maximal effort). Participants were allowed to choose from a variety of aerobic activities, including running, cycling, rowing, swimming, *etc*.

### 2.5. Laboratory Testing Procedures

All testing was performed between 0600 and 0900, following a 12-h fast and 48-h abstinence from caffeine and alcohol intake, and 48–72 h after the last exercise session to eliminate the acute effects of the last bout of exercise. These tests were performed at Weeks 0 and 13 and performed by the same investigators for the pre- and post-intervention testing.

### 2.6. Cardiometabolic Biomarkers

*Blood lipids and C-reactive protein:* A 12-h fasted venous blood sample (~20 mL) was obtained (Week 0 and Week 13). Blood was collected into EDTA-coated vacutainer tubes and centrifuged (Hettich Rotina 46R5) for 15 min at 2500 rpm at 4 °C. Upon separation, plasma was stored at −70 °C in aliquots until analyzed. Plasma C-reactive protein and insulin concentrations were determined using commercially available ELISA kits (Millipore, Billerica, MA, USA). Total cholesterol (TC), high-density lipoprotein cholesterol (HDL-C), and triglycerides (TRGs) were assessed using the Cholestech LDX blood analysis system (Hayward, CA, USA). The test–retest intraclass correlation (r) and coefficient of variation (CV) in our laboratory with *n* = 15 are as follows: TC and HDL-C (mg/dL); *r* = 0.95, CV = 3.2%; *r* = 0.97, CV = 5.3%, respectively.

*Heart rate and blood pressure:* Resting heart rate and systolic and diastolic blood pressure (BP) were obtained in the supine position as previously described [2]. Heart rate and BP were obtained following a minimum of 10 min of quiet resting.

*Arterial function:* Vascular health was assessed using pulse contour analysis (augmentation index) and pulse wave velocity (Arteriograph, version 1.10.0.1, TensioMed Kft., Budapest, Hungary). Augmentation index was determined by the following formula:

$$Aix\;\left(100\%\right)=\left(P_{2}-\;P_{1}\right)/PP\;\times \;100$$

where P_1_ is the early (direct) wave’s amplitude; P_2_ is the late (reflected) systolic wave’s amplitude; and PP equals the pulse pressure.

The aortic pulse wave velocity (PWVao) was determined by the wave reflection generated from the early direct pulse wave as it is reflected back from the aortic bifurcation. Return time (RT) is determined by measuring the time interval between peaks from the early direct (P_1_) and reflected late (P_2_) systolic waves. The PWVao calculations were measured using the distance from the upper edge of the pubic bone to the sternal notch (Jugulum-Symphisis¼), as this provides the closest approximation of the actual aortic length. PWVao was calculated with the following formula:

PWVao (m/s) =[Jug−Sy (m)]/[(RT/2)(s)]

where RT is return time; and Jug–Sy is the aortic distance (Jugulum–Symphisis). The test–retest intraclass correlation (r) coefficient of variation (CV) in our laboratory with *n* = 10 are as follows: PWV and RT; *r* = 0.94, CV = 11.2%; *r* = 0.90, CV = 12.0%, respectively.

### 2.7. Resting Energy Expenditure (REE)

Resting metabolic rate (RMR) was measured (Weeks 0 and 13) using the ventilated hood technique (ParvoMedic; analyzed via True One 2400 software). Specifically, participants arrived at the Human Nutrition and Metabolism Laboratory between 0600 and 0800 with minimal physical movement and fasted for 10–12 h. Following 20 min of relaxed supine lying, REE was measured for 30 min in a darkened, temperature controlled room. The test–retest intraclass correlation (r) and coefficient of variation (CV) in *n* = 14 are as follows: RMR (Kcal/min); *r* = 0.92, 4.2%, respectively.

### 2.8. Total and Regional Body Composition

Anthropometric and body composition measurements were obtained at Weeks 0 and 13. At each visit, body weight was measured with a standard digital scale (Befour Inc. Cedarburg, WI, USA), height was measured using a stadiometer, and waist circumferences were measured with a standard tape measure placed at the area with the smallest circumference between the rib cage and the iliac crest. As described previously, body composition was assessed by dual energy X-ray absorptiometry (iDXA; Lunar iDXA; GE Healthcare, Madison, WI, USA; analyzed using encore software version 13.6) for total body adiposity, % body fat, lean body mass, visceral adipose tissue (VAT), and regional abdominal adiposity [2]. The test–retest intraclass correlation (r) and coefficient of variation (CV) for body composition analysis using iDXA in our laboratory with *n* = 12 are as follows: LBM and FM; *r* = 0.99, CV = 0.64%; *r* = 0.98, CV = 2.2%, respectively. For regional abdominal body composition analysis, they are as follows: %FAT: *r* = 0.99, CV = 2.4%.

### 2.9. Dietary Intake and Feelings of Hunger and Satiety

Throughout the intervention, subjects maintained a daily food log that included all food and beverages consumed each day, including meal timing. To further verify compliance, food intake was analyzed from a representative 3-day period at Weeks 0 and 12 using Food Processor SQL Edition (version 10.12.0, 2012; ESHA Research, Salem, OR, USA) [2]. All dietary analyses were performed by the same technician. Visual analog scales (VAS’s) were administered at baseline and Week 13 to evaluate the effects of the lifestyle interventions on hunger, satiation, and a desire to eat [2].

### 2.10. Physical Performance Assessments

Following a familiarization session for all testing procedures, physical performance outcomes were assessed at Weeks 0 and 13 at the same time of day and completed over a 2-day period. For example, aerobic power (5-km TT), muscular endurance (sit-ups/push-ups), flexibility (sit and reach), and balance (standing stork balance) were completed on Day 1, whereas upper and lower body strength (bench press/leg press) and power tests (squat jumps/bench throws) and vertical jumps were completed on Day 2 (See below).

*Upper Body Muscular Endurance*. Upper body muscular endurance was assessed with timed push-ups in 1 min. Women started in the plank position balancing on the knees with arms extended and hands placed under the shoulders. A successful push-up was defined as lowering the body so that elbows reached 90° followed by a return to the starting plank position. Participants were asked to perform as many push-ups as possible within 60 s in a continuous pattern with no more than two seconds of rest between repetitions.

*Core Muscular Endurance*. Timed sit-ups were performed in the supine position with arms folded across the chest, knees bent at 90°, and feet flat on the ground and supported by a research team member. A successful sit-up required participants to curl up to a 90° position (vertical) to the floor and then return to the starting position. The sit-up action was continuous, with a rest duration of no more than 2 s allowed between repetitions. Participants were instructed to perform as many sit-ups as possible in 60 s.

*Standing Balance*. Postural balance was assessed with the stork balance test. While in the standing position, participants were instructed to balance on the dominant leg with the heel lifted off the ground and the non-dominant knee flexed to 90°, with the foot placed gently against the inside of the dominant knee. Hands were placed on the hips at the level of the iliac crests. The trial ended when the heel of the dominant leg touched the floor, the hands came off of the hips, or the non-dominant foot was removed from the dominant standing leg. Participants were provided three attempts and the best time was recorded for analysis.

*Flexibility*. Lower back and hamstring flexibility were assessed with the sit-and-reach test. This was administered using a standard sit-and-reach box (Lafayette Instrument Company, Lafayette, IN, USA), following a standard technique. The maximal distance reached of 3 trials was recorded.

*5-km Cycle Ergometer Time Trial.* Subjects arrived to the laboratory for performance testing sessions having consumed a standardized meal (PRISE, IsaLean bar; CON, granola bar) 1 h prior. Before the time trial began, seat and handle bar lengths, height, and tilt were adjusted according to each subject’s preferences. Each adjustment was recorded and used for the post-test (Week 13). Following a 5–7 min warm-up at 60% of heart rate reserve (HRR) on the Velotron Dynafit Pro cycle ergometer (Racermate, CompuTrainer 3D Software, Version 1, Seattle, WA, USA), participants completed a 5-km time trial (5-km TT) as fast as possible. Pedaling cadence and gear ratio were selected freely by the participant during each ride (Weeks 0 and 13). Subjects were permitted to drink water, if needed (*ad libitum*). Total time to complete the time trial and mean and max watts were all recorded. HR and blood pressure were recorded every five minutes during the time trial immediately upon finishing and 5 and 10 min after completion.

*Upper and Lower Body Maximal Strength*. Measures of one repetition maximal strength (1 RM) of the upper and lower body were assessed via the bench (barbell) and leg press, respectively, as previously described [7]. The test–retest intraclass correlation (r) and coefficient of variation (CV) in *n* = 15 are as follows: chest 1-RM and leg 1-RM; *r* = 0.99, CV = 1.6%; *r* =0.99, CV = 2.7%, respectively.

*Upper and Lower Body Maximal Force and Power*. Following 1 RM’s of the bench and leg press, dynamic maximal force and power of the upper and lower body were assessed with bench throws (BTs) and jump squats (JS’s), respectively, using the Ballistic Measurement System (Innervations Inc., Muncie, IN, USA) interfaced with a commercial smith rack. Prior to performing the tests participants were provided instructions on how to perform the tests safely and with proper techniques. During the familiarization process, subjects performed 3–5 un-weighted practice trials for the BTs and JS’s. For the JS’s, participants performed three consecutive repetitions with the barbell loaded to 30% of their predetermined IRM for the leg press. Participants began the JS’s in the standing position with feet slightly wider than hip width apart and the loaded barbell across the upper trapezius muscles. When instructed, they lowered into the squat position until 90° of knee flexion was achieved, then jumped as high as possible, and landed with bent knees. Immediately upon landing, without pause, participants repeated the same upward jumping movement for a total of three maximal JS’s in succession.

For the bench throws (BTs), participants followed identical familiarization procedures as the JS’s by performing 3–5 un-weighted practice trials lying supine on a bench with hands positioned on the barbell slightly wider than shoulder width apart and arms fully extended. The bar was then loaded with 20% of the 1 RM of the bench press. To initiate the BTs, subjects lowered the barbell to the chest just above the distal end of the sternum and were instructed to explosively push and then release the barbell with the intent to project the barbell as high as possible. Participants caught the bar on its descent and immediately, without pause, initiated another maximal BT until 3 successive repetitions were completed. Throughout both the JS and BT tests, spotters were present on both sides of the barbell to provide verbal encouragement and ensure safety of the participants. The physical performance variables measured and used for analysis were mean and peak power (watts) taken as an average of the three repetitions.

### 2.11. Statistical Analysis

Statistical analysis was performed using SPSS software (Ver. 23; IBM). A 2 × 2 repeated measures ANOVA was performed to assess differences between groups (PRISE *vs.* CON) and time (pre *vs.* post) to determine main effects and interactions. *Post hoc* comparisons (Bonferroni) were performed to determine whether there was an interaction with the addition of between-group independent samples *t*-tests at the pre- and post-time points. One-tailed tests were utilized for this study based on our previous investigation showing improved body composition metrics following PRISE training [2], and the significance was set at *p* < 0.05. All values are reported as means ± standard deviation unless stated otherwise. Before the start of the study, sample size was determined through power analysis (80%) based on the major outcome variables (muscular strength, body composition, and arterial function). This analysis determined that *n* = 12 was required to detect significant differences between groups. Absolute changes in muscular strength (kg), body weight (kg), and arterial function change were calculated.

## 3. Results

### 3.1. Participant Characteristics and Compliance

The participant characteristics are presented in Table 1. Prior to the intervention, all variables in each outcome domain (physical performance, cardiovascular health, body composition, diet, and metabolic profile) were not different between groups. Three participants in the PRISE group were excluded from analysis due to non-compliance to the diet and/or exercise routine, resulting in an 80% adherence rate for both the nutrition and exercise interventions.

### 3.2. Muscular Fitness and Exercise Performance

**Figure 2:**
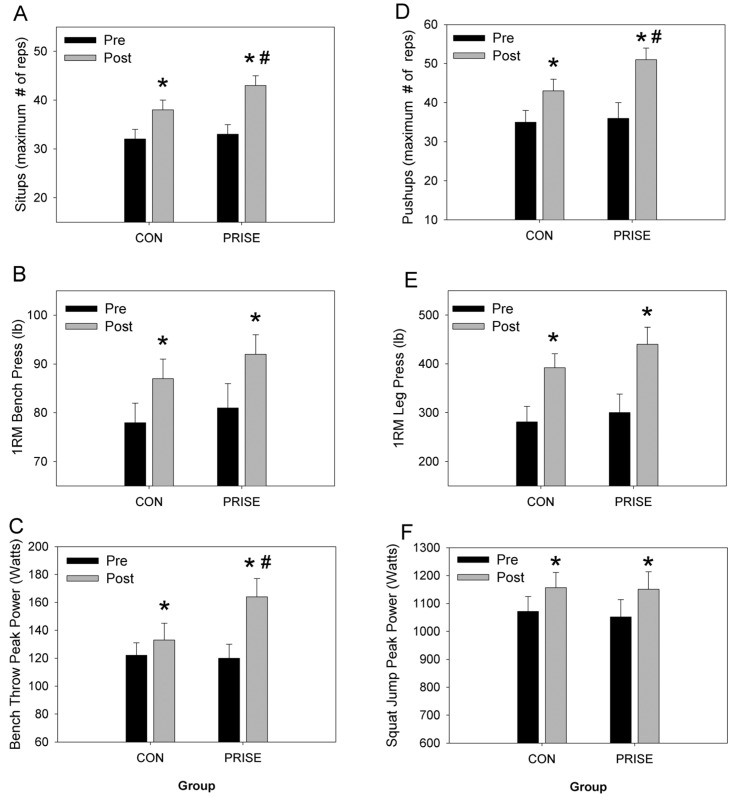
Muscle function parameters at baseline (pre) and after 12 weeks (post) between PRISE and CON. * *p* < 0.05 pre *vs*. post training, # *p* < 0.05 group difference in training response. CON, normal protein; PRISE, protein-pacing. Mean ± SD.

By design, each of the fitness and performance outcomes was improved following the interventions. Specifically, core (abdominal sit-ups) and upper body muscular endurance (push-ups) were improved (training effect, *p* < 0.01, Figure 2A,D) and to a significantly greater extent in the PRISE group (interaction, *p* < 0.01). Upper and lower body maximal strength, assessed via 1-RM bench press and leg press, respectively, significantly improved (*p* < 0.01, Figure 2B,E), and no group differences were found. Likewise, upper (bench throws) and lower (squat jumps) body muscle power significantly improved as a result of the training (*p* < 0.05, Figure 2C,F), and upper body power increased to a greater extent in the PRISE group (interaction, *p* < 0.05, Figure 2C).

Flexibility, as assessed by the sit-and-reach test, significantly (*p* < 0.05) improved following the intervention (CON: 37 ± 2 *vs.* 40 ± 2; PRISE: 34 ± 2 *vs.* 37 ± 2 cm, pre- *vs.* post-intervention, respectively), though no differences were found between groups. Balance, assessed with the stork stand test, significantly (*p* < 0.05) improved following the intervention, but no differences were found between groups (CON: 6.4 ± 0.8 *vs.* 9.8 ± 2.9 s; PRISE: 3.2 ± 1.0 *vs.* 10.7 ± 3.2 s, pre- *vs.* post-intervention, respectively). Lastly, aerobic power, as assessed by time to complete a 5-km cycling time trial, significantly (*p* < 0.05) improved following the training (CON: 621 ± 11 *vs.* 586 ± 9 s; PRISE: 613 ± 12 *vs.* 592 ± 10 s, pre- *vs.* post-intervention, respectively); however, no differences were found between groups.

### 3.3. Cardiovascular Health

**Figure 3:**
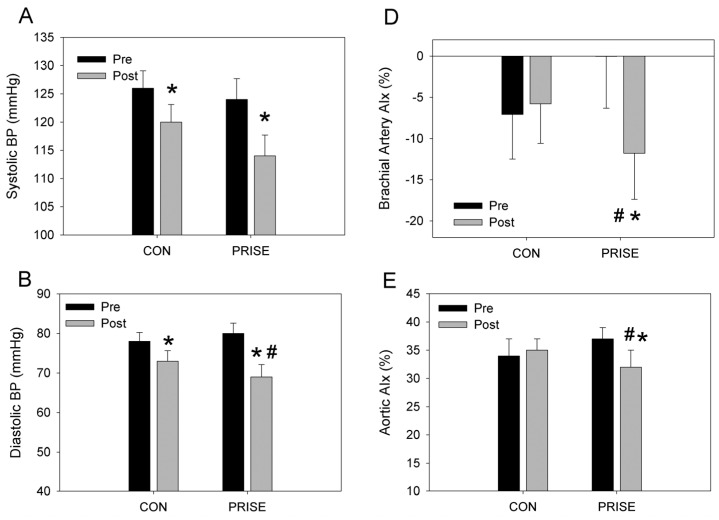

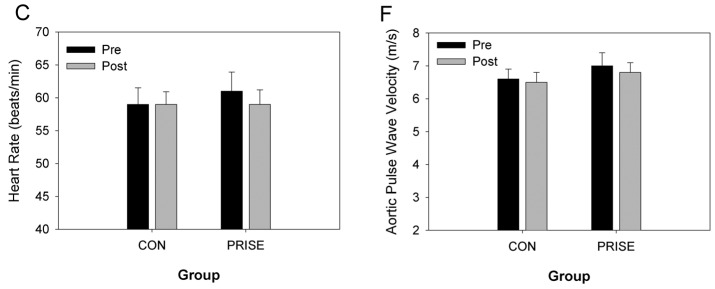
Cardiovascular responses to 12 weeks of PRISE and CON. * *p* < 0.05 pre *vs.* post training, # *p* < 0.05 group difference in training response. CON, normal protein; PRISE, protein-pacing. Mean ± SD.

Both systolic and diastolic blood pressures significantly improved following the exercise intervention (*p* < 0.05, Figure 3A,B), though diastolic blood pressure fell to a greater degree in the PRISE group (interaction, *p* < 0.05). Resting heart rate was unaffected by the exercise intervention or by the protein supplementation (*p* > 0.05, Figure 3C). Augmentation index of both the brachial artery and aorta improved following the training intervention (*p* < 0.05), an effect that was more pronounced in the PRISE group (interaction, *p* < 0.01, Figure 3D,E). Aortic pulse wave velocity and return time were not significantly impacted by the intervention in either group (*p* > 0.05, Figure 3F). The assessment of circulating C-reactive protein was unaffected by the training in either group (CON: 0.47 ± 0.85 *vs.* 0.42 ± 0.57 µg/mL; PRISE: 0.50 ± 1.2 *vs.* 0.72 ± 1.9 µg/mL, pre- *vs.* post-intervention, respectively).

### 3.4. Body Composition

Body composition significantly improved in both groups following the training protocol, though no interactions were observed between groups. Independent of changes in body weight, significant improvements were observed in body composition. Of particular note was the significant increase in percent lean body mass and decrease in abdominal and hip fat following the intervention in both groups (Table 2).

### 3.5. Diet, Satiety, and Hunger

At baseline, all participants met recommended daily intakes and were not different between groups (Table 3). By design, the PRISE group consumed significantly more protein in absolute (grams) and relative (grams/kg body weight) terms (interaction, *p* < 0.05). The PRISE women experienced a reduction, whereas CON women showed an increase in self-reported VAS question “How much food do you feel like you could eat right now?” (interaction, *p* < 0.05). All other dietary factors remained constant across the intervention and similar between groups (Table 3).

### 3.6. Metabolic Profile

The exercise training protocol reduced resting metabolic rate (*p* < 0.05) by ~5%, with no group effect (Table 4). Although fasting blood glucose increased following the intervention in both groups, it remained within normal, healthy levels. Total plasma cholesterol levels declined in both groups (*p* = 0.04), and insulin remained unchanged from baseline (Table 4).

## 4. Discussion

The aim of this study was to determine the effect of a 12-week protein-pacing (P) diet (PRISE, 5–6 meals/day at 2.0 g/kg·BW/day) compared to a normal protein intake (CON, 5–6 meals/day at 1.0 g/kg·BW/day), both of which included a multimodal RISE training program (Resistance, Interval, Stretch and Endurance) on physical performance (muscular fitness; strength, power, flexibility; and aerobic fitness), cardiovascular measures, and body composition in exercise-trained, healthy women. The main findings of the current study are as follows: (1) The RISE protocol elicited significant improvements in performance (5-km TT, upper and lower body maximal strength and power, flexibility, and balance), and some of these improvements were enhanced in the PRISE group (2.0 g/kg/day), specifically abdominal and upper body strength and power; (2) in terms of the effects of RISE training on cardiovascular outcomes (systolic and diastolic blood pressure as well as augmentation index, AIx) and body composition (% fat, fat free mass, fat mass, abdominal fat, and hip fat), all improved with training, and the PRISE group exhibited greater reductions in DBP and Aix; and (3), following the intervention, the PRISE group exhibited an enhanced satiety compared to the CON group (1.0 g/kg·BW/day).

Collectively, these results demonstrate, for the first time, that the multimodal RISE protocol improves all aspects of performance (muscle strength, power, flexibility, balance, and endurance) in active healthy females. In addition, adding a protein-pacing dietary intake pattern (5–6 meals/day at 2.0 g/kg·BW/day) confers additional benefit from training, enhancing the increases in upper body muscle strength and power, abdominal strength, as well as eliciting greater reductions in diastolic blood pressure and augmentation index in active women.

### 4.1. Fitness and Performance Outcomes

Previously, the multimodal RISE training protocol was used in overweight/obese men and women, targeting improvements in body composition and cardiometabolic risk reductions [2]. Thus, it remained unanswered whether RISE may enhance physical performance outcomes. Research on concurrent strength and endurance training has revealed that either endurance capacity [8] or muscle strength [9] may be compromised due to conflicting physiological mechanisms or perhaps the reallocation of training volume or overtraining. In the current study, there was no apparent blunting of improvements in endurance performance (5-km TT), muscle strength (1 RM), muscle power (jump squat or bench throw), flexibility (sit and reach), balance (stork stand), or muscle endurance (maximum # of push-ups and sit-ups). Thus, we contend that a multimodal training paradigm is not detrimental to fitness-specific performance gains and may actually be complementary for facilitating improvements, possibly promoting the avoidance of injury and symptoms of over training (*i.e.*, burn out).

Ingestion of whey protein or protein supplements is highly prevalent in both the athletic and recreational populations, ranging from 13% to 75% [10,11,12]. In recreational athletes, Cantarow *et al.* [10] reported that 75% of males consumed protein supplements. While lower than males, 50% of females reported the use of a protein supplement. Thus, understanding the effects of high dietary protein on performance is warranted. Some studies have demonstrated that increasing protein intake above RDA levels can positively influence body composition and/or athletic performance measures. Our data are in agreement with these studies. Despite this, the majority of studies suggest an acute benefit to muscle protein synthesis [13,14,15,16,17] and/or recovery [18,19]. In support of this, our findings are in agreement with those of others showing a training-induced improvement in performance outcomes [4,5,20], and we extend these findings to demonstrate this in recreationally active healthy women. A recent review on the topic reported that protein ingestion of 0.4 g/kg/meal optimally stimulates muscle protein synthesis [3]. Interestingly, our protein intake per meal in the current study was 0.41 g/kg/meal, which may have partly accounted for the significant improvement in physical performance outcomes in PRISE compared to CON. The lack of differences in body composition between groups despite the maximally stimulating dose of protein ingestion per meal in the PRISE group suggests that body composition changes may be delayed compared to muscular performance adaptations to the higher protein per meal ingestion in women. As such, a longer intervention may be required to detect changes in body composition. Work in mice has suggested that ingestion of high dietary protein (whey) increased muscle strength and endurance [21]. Indeed, in humans, supplemental protein ingestion has improved running endurance performance by 4 km over a one-week intensive training camp [20] and may prevent decline during such training [22]. On the contrary, another study found that acute protein ingestion [23] did not improve aerobic performance. However, participants with the lowest level of fitness/performance were found to benefit from the protein ingestion.

In the current study, we found, as expected, that the RISE training improved every aspect of performance (muscle strength, power, balance, flexibility, endurance performance); additionally, protein-pacing resulted in a synergistic effect, further improving upper body and abdominal muscle strength and endurance (maximum # of push-ups and sit-ups, respectively) and upper body muscle power (bench throw) (Figure 2) in previously active women. Most previous studies investigating the potential performance benefits of protein ingestion have almost exclusively focused on men [17,20,22,23,24]. Thus, the current study is in agreement with previous investigations documenting an increased protein intake and enhanced performance outcomes [5,20]. It remains to be seen if such protein supplementation might extend to other populations, such as highly-trained athletes, and whether greater amounts of dietary protein (2.0–4.0 g/kg·BW/day) are warranted.

### 4.2. Cardiovascular Health

Previous investigations of training on vascular health have revealed positive responses to either resistance, interval [25], flexibility (e.g., yoga) [26], or endurance exercise training [25], and very few have combined these training modalities [27,28]. Acute ingestion of milk and/or whey proteins alone has been demonstrated to improve vascular health or factors contributing to CVD risk [29,30,31]. In the current study, we demonstrate that 12 weeks of a mixed modality training program targeting multiple aspects of fitness (muscular endurance, strength and power, flexibility, aerobic power, and balance) resulted in significant reductions in both systolic and diastolic blood pressure. Specifically, on average, the groups reduced both systolic and diastolic blood pressure by ~8 mmHg (Figure 3). Such changes are known to significantly reduce risk of coronary heart disease events and stroke, by approximately 25% and 36%, respectively [32]. It is also important to note that the PRISE group experienced a tendency for a greater reduction in systolic (∆10 *vs.* ∆6 mmHg, PRISE *vs.* CON) and a significantly greater reduction in diastolic blood pressure (∆11 *vs.* ∆5 mmHg, PRISE *vs.* CON), which, again, might translate into a meaningful reduction in risk for CV related events. Augmentation index (AIx), but not pulse wave velocity, corroborates the blood pressure findings, indicating a significant reduction with training, which was enhanced in the PRISE group (Figure 3) and likely translates into a reduction in CV risk [33]. Taken together, these findings suggest that the multimodal RISE training improves vascular health, which can be further enhanced with protein-pacing intake.

As exercise paradigms shift and new guidelines are developed, it is important to understand how each fitness component may influence vascular health and the importance of performing more than one type of exercise training (RISE protocol). In light of previous investigations that suggest resistance training elevates vascular stiffness [34], the current study highlights that, using a multimodal training protocol, central pulse wave velocity was not altered, and, in fact, augmentation index was reduced. In combination with the reductions in diastolic blood pressure, this reduction in AIx is suggestive of a training-induced reduction in peripheral resistance, which was enhanced with protein-pacing (PRISE).

### 4.3. Body Composition

Our previous work in overweight and obese men and women [2] demonstrated that the PRISE protocol elicited a significantly greater improvement in lean body mass, reductions in fat mass, and visceral adipose tissue over a protein-pacing diet with and without a concomitant resistance training program. Here, we demonstrate that the RISE protocol enhances body composition (increases lean body mass, decreases total and abdominal fat mass) in healthy, normal-weight women. The prior investigation compared protein-pacing alone with protein-pacing with resistance training, or with the RISE protocol (PRISE) [2], and showed that, in overweight/obese individuals, PRISE was more efficacious in improving body composition than protein-pacing combined with resistance training, or protein-pacing alone [2]. In the current study, RISE training significantly improved body composition (total and abdominal fat mass, hip fat, and lean body mass) in normal-weight women, regardless of protein intake (Table 2).

While increased protein [35] and/or increased meal frequency [1] alone, or when combined with exercise [3,36], have been shown to improve body composition in normal- and overweight adults, we did not see additional benefit of protein-pacing on body composition in active normal-weight women performing RISE training. However, recent work by Antonio *et al.* [37] indicated that high protein intake in combination with heavy resistance training did elicit additional improvement in body composition, namely, a greater reduction in fat mass and % body fat. However, it is important to note that the definition of high protein in that study [37] was 3.4 g/kg·body weight/day *versus* the 2.0 g/kg·BW/day used in the current study. While the recommended dietary intake for protein is 0.8 g/kg·BW/day, Antonio *et al.* assigned participants to 3.4 g/kg·BW/day and observed no adverse effects on metabolic profile, including markers of kidney function with intakes as high as 4.4 g/kg·BW/day [38]. Thus, it is possible that additional protein intake beyond 2.0 g/kg·BW/day may provide additional body composition benefit over the RISE training alone and warrants further investigation.

### 4.4. Hunger Ratings and Dietary Intake

In the current study we find that feelings of satiation (“*How much food do you feel you could eat right now*?”) were significantly enhanced in the PRISE group but not the CON group following the intervention (Table 3). While the other indicators of satiety or hunger were not significantly different between groups, this finding of improved satiety is supported by previous work from our lab and by others that also suggest increased satiety with increased protein intake [39,40]. By design, macronutrient intake, specifically protein intake, was different between groups and on target for the protein goals of both the control (CON, 1 g/kg·BW/day) and the PRISE (2 g/kg·BW/day) groups (Table 3). All other dietary factors were not different between groups (with the exception of dietary cholesterol intake). Thus, any differences observed between the groups were likely attributed to the PRISE and warrants further investigation.

### 4.5. Metabolic Profile

Prior investigations of high dietary protein intake suggest that elevated protein intake has the potential to either acutely [30] or chronically improve cardiometabolic profile [31]. Though, it is important to note that the magnitude of protein ingestion (g/kg·BW) as well as the population studied (healthy *vs.* disease) likely play a role in whether PRISE alters metabolic profile and the degree to which it is improved. The current study demonstrated a slightly improved metabolic efficiency (~5% reduction in RMR), which corroborates previous investigations [41]. Additionally, the reduction in total cholesterol supports previous work demonstrating an improved cardiovascular risk profile in response to exercise training [42].

## 5. Conclusions

The multimodal RISE training protocol improves multiple aspects of performance (core and upper body maximal strength and power, aerobic power, balance, and flexibility), cardiovascular health, and body composition. Furthermore, inclusion of protein-pacing (P, 2.0 g/kg·BW/day) confers additional benefit in core and upper body strength and power, as well as cardiovascular health (DBP and AIx) in active normal-weight women. The results from this study provide compelling evidence that increasing dietary protein intake to more than twice the current RDA may further augment the training-induced adaptations to multimodal exercise training programs with additional cardiovascular benefits.

## Figures and Tables

**Table 1:** Baseline subject characteristics (*N* = 27).

	CON (*n* = 15)	PRISE (*n* = 12)
Age (year)	42 ± 7	42 ± 9
Height (cm)	166 ± 6	165 ± 7
Weight (kg)	65 ± 9	65 ± 7
Body Mass Index (kg/m^2^)	24 ± 3	24 ± 2
Systolic Blood Pressure (mmHg)	126 ± 11	127 ± 18
Diastolic Blood Pressure (mmHg)	78 ± 8	82 ± 12
Pulse Pressure (mmHg)	48 ± 6	44 ± 6
Heart Rate (beats/min)	59 ± 11	61 ± 7
Total Cholesterol (mg/dL)	184 ± 22	185 ± 37
HDL Cholesterol (mg/dL)	68 ± 17	67 ± 11
LDL Cholesterol (mg/dL)	97 ± 21	107 ± 25
Triglycerides (mg/dL)	88 ± 44	88 ± 43
Glucose (mg/dL)	81 ± 7	81 ± 6

CON: normal protein (5–6 meals/day at 1.0 g/kg BW/day); PRISE: protein-pacing (5–6 meals/day at 2.0 g/kg·BW/day); HDL: High Density Lipoprotein; LDL: Low density lipoprotein. Data are means ± standard deviation.

**Table 2:** Changes in body composition pre- and post-intervention.

		Pre	Post
Body Weight (kg)	CON	65.4 ± 9.4	64.8 ± 9.5
	PRISE	64.8 ± 7.3	64.6 ± 7.3
Body Fat (%) *	CON	31.9 ± 6.6	30.9 ± 6.2
	PRISE	30.8 ± 6.1	29.5 ± 7.0
Fat Mass (kg) *	CON	20.3 ± 6.3	19.6 ± 6.1
	PRISE	19.3 ± 5.4	18.6 ± 5.9
Fat Free Mass (kg) *	CON	44.8 ± 5.2	45.2 ± 5.1
	PRISE	45.0 ± 4.1	45.9 ± 4.3
Lean Body Mass (kg) *	CON	42.3 ± 5.0	42.7 ± 4.8
	PRISE	42.6 ± 4.0	43.5 ± 4.2
% Lean Body Mass (%) *	CON	65.2 ± 6.7	66.5 ± 5.9
	PRISE	66.1 ± 5.6	67.7 ± 6.2
Abdominal Fat (%) *	CON	30.8 ± 10.6	29.0 ± 10.0
	PRISE	28.5 ± 9.5	26.7 ± 11.5
Hip Fat (%) *	CON	35.9 ± 5.6	34.7 ± 5.1
	PRISE	35.9 ± 6.4	33.7 ± 7.2

CON: normal protein (5–6 meals/day at 1.0 g/kg BW/day); PRISE: protein-pacing (5–6 meals/day at 2.0 g/kg BW/day). Data are means ± standard deviation. *: denotes significant effect of intervention (pre *vs*. post).

**Table 3:** Diet, satiety, and hunger ratings pre- and post-intervention.

		Pre	Post
Caloric Intake (kcal/day)	CON	1631 ± 285	1608 ± 282
	PRISE	1662 ± 149	1756 ± 171
Fat Intake (g/day)	CON	58 ± 15	56 ± 20
	PRISE	58 ± 17	53 ± 19
Carbohydrate Intake (g/day)	CON	188 ± 55	193 ± 53
	PRISE	172 ± 63	177 ± 42
Protein Intake (g/day) #	CON	77 ± 12	69 ± 10
	PRISE	75 ± 23	131 ± 16
Protein Intake (g/kg BW/day) #	CON	1.2 ± 0.2	1.1 ± 0.1
	PRISE	1.2 ± 0.4	2.0 ± 0.1
Cholesterol Intake (mg/day) #	CON	212 ± 115	169 ± 99
	PRISE	170 ± 139	286 ± 125
Sodium Intake (mg/day)	CON	1856 ± 920	1993 ± 639
	PRISE	1816 ± 594	1822 ± 620
Fiber Intake (g/day)	CON	21 ± 7	27 ± 11
	PRISE	19 ± 7	23 ± 8
How hungry are you feeling? (0–100)	CON	40 ± 17	45 ± 17
	PRISE	42 ± 22	44 ± 23
How full do you feel? (0–100)	CON	28 ± 18	35 ± 15
	PRISE	24 ± 21	34 ± 23
How much food could you eat? (0–100) #	CON	44 ± 11	54 ± 10
	PRISE	49 ± 20	43 ± 19
What is your desire to eat? (0–100)	CON	42 ± 14	47 ± 17
	PRISE	41 ± 33	43 ± 27

CON: normal protein (5–6 meals/day at 1.0 g/kg BW/day); PRISE: protein-pacing (5–6 meals/day at 2.0 g/kg BW/day). Data are means ± standard deviation. #: denotes significant interaction of group (CON; 1 g/kg of body weight) *vs.* (PRISE; 2 g/kg of body weight).

**Table 4:** Metabolic profile pre- and post-intervention.

		Pre	Post
Resting Metabolic Rate (kcal/day) *	CON	1385 ± 195	1322 ± 147
	PRISE	1453 ± 147	1367 ± 98
Respiratory Exchange Ratio	CON	0.80 ± 0.04	0.80 ± 0.05
	PRISE	0.80 ± 0.05	0.79 ± 0.04
CHOox (%)	CON	34 ± 20	33 ± 17
	PRISE	32 ± 16	30 ± 14
FATox (%)	CON	66 ± 20	67 ± 17
	PRISE	68 ± 16	70 ± 14
Fasting Blood Glucose (mg/dL) *	CON	81 ± 7	83 ± 5
	PRISE	81 ± 6	84 ± 6
Insulin (uU/mL)	CON	2.7 ± 1.2	2.5 ± 0.5
	PRISE	2.5 ± 0.4	2.5 ± 0.4
Total Cholesterol (mg/dL) *	CON	185 ± 22	182 ± 21
	PRISE	185 ± 37	175 ± 27
HDL Cholesterol (mg/dL)	CON	68 ± 17	69 ± 13
	PRISE	67 ± 11	67 ± 12
LDL Cholesterol (mg/dL)	CON	93 ± 21	94 ± 25
	PRISE	107 ± 25	96 ± 27
Total Cholesterol/HDL	CON	2.9 ± 0.9	2.7 ± 0.6
	PRISE	2.6 ± 0.4	2.6 ± 0.3
Triglycerides (mg/dL)	CON	92 ± 40	87 ± 31
	PRISE	88 ± 43	89 ± 28

CON: normal protein (5–6 meals/day at 1.0 g/kg BW/day); PRISE: protein-pacing (5–6 meals/day at 2.0 g/kg BW/day). CHOox: relative contribution of carbohydrate to energy expenditure; FATox: relative contribution of fat to energy expenditure; HDL: High Density Lipoprotein; LDL: Low density lipoprotein. Data are means ± standard deviation. * denotes significant effect of intervention (pre *vs.* post).

## Supplementary Materials

The following are available online at http://www.mdpi.com/2072-6643/8/6/332/s1.

## Abbreviations

The following abbreviations are used in this manuscript:

PRISE	protein-pacing, resistance, interval, stretching, endurance training
CON	control
AIx,	augmentation index
PWV	pulse wave velocity
SUs	sit-ups
PUs,	push-ups
5-km TT	five kilometer time-trial
BT’s	bench throws
JS’s	jump squats
DBP	diastolic blood pressure
SBP	systolic blood pressure
TC	total cholesterol
HDL-C	high density lipoprotein cholesterol;
LDL-C	low density lipoprotein cholesterol
TRGs	triglycerides
GLU	glucose
REE	resting energy expenditure

## References

[1] Arciero P.J., Ormsbee M.J., Gentile C.L., Nindl B.C., Brestoff J.R., Ruby M. (2013). Increased protein intake and meal frequency reduces abdominal fat during energy balance and energy deficit. Obesity.

[2] Arciero P.J., Baur D., Connelly S., Ormsbee M.J. (2014). Timed-daily ingestion of whey protein and exercise training reduces visceral adipose tissue mass and improves insulin resistance: The prise study. J. Appl. Physiol..

[3] Morton R.W., McGlory C., Phillips S.M. (2015). Nutritional interventions to augment resistance training-induced skeletal muscle hypertrophy. Front. Physiol..

[4] Volek J.S., Volk B.M., Gomez A.L., Kunces L.J., Kupchak B.R., Freidenreich D.J., Aristizabal J.C., Saenz C., Dunn-Lewis C., Ballard K.D. (2013). Whey protein supplementation during resistance training augments lean body mass. J. Am. Coll. Nutr..

[5] Wilborn C.D., Taylor L.W., Outlaw J., Williams L., Campbell B., Foster C.A., Smith-Ryan A., Urbina S., Hayward S. (2013). The effects of pre- and post-exercise whey *vs.* Casein protein consumption on body composition and performance measures in collegiate female athletes. J. Sports Sci. Med..

[6] Arciero P.J., Miller V.J., Ward E. (2015). Performance enhancing diets and the prise protocol to optimize athletic performance. J. Nutr. Metab..

[7] Arciero P.J., Gentile C.L., Martin-Pressman R., Ormsbee M.J., Everett M., Zwicky L., Steele C.A. (2006). Increased dietary protein and combined high intensity aerobic and resistance exercise improves body fat distribution and cardiovascular risk factors. Int. J. Sport Nutr. Exerc. Metab..

[8] Glowacki S.P., Martin S.E., Maurer A., Baek W., Green J.S., Crouse S.F. (2004). Effects of resistance, endurance, and concurrent exercise on training outcomes in men. Med. Sci. Sports Exerc..

[9] Bell G.J., Syrotuik D., Martin T.P., Burnham R., Quinney H.A. (2000). Effect of concurrent strength and endurance training on skeletal muscle properties and hormone concentrations in humans. Eur. J. Appl. Physiol..

[10] Cantarow M.E., Livermore A.A., McEntee K.B., Brown L.S. (2015). Differences in the use of protein supplements and protein-rich food as seen among us recreational athletes. Top. Clin. Nutr..

[11] Lieberman H.R., Marriott B.P., Williams C., Judelson D.A., Glickman E.L., Geiselman P.J., Dotson L., Mahoney C.R. (2015). Patterns of dietary supplement use among college students. Clin. Nutr..

[12] Heikkinen A., Alaranta A., Helenius I., Vasankari T. (2011). Dietary supplementation habits and perceptions of supplement use among elite finnish athletes. Int. J. Sport Nutr. Exerc. Metab..

[13] Moore D.R., Soeters P.B. (2015). The biological value of protein. Nestle Nutr. Inst. Workshop Ser..

[14] Arentson-Lantz E., Clairmont S., Paddon-Jones D., Tremblay A., Elango R. (2015). Protein: A nutrient in focus. Appl. Physiol. Nutr. Metab..

[15] Churchward-Venne T.A., Murphy C.H., Longland T.M., Phillips S.M. (2013). Role of protein and amino acids in promoting lean mass accretion with resistance exercise and attenuating lean mass loss during energy deficit in humans. Amino Acids.

[16] Phillips S.M. (2014). A brief review of critical processes in exercise-induced muscular hypertrophy. Sports Med..

[17] Phillips S.M., Hartman J.W., Wilkinson S.B. (2005). Dietary protein to support anabolism with resistance exercise in young men. J. Am. Coll. Nutr..

[18] Buckley J.D., Thomson R.L., Coates A.M., Howe P.R., DeNichilo M.O., Rowney M.K. (2010). Supplementation with a whey protein hydrolysate enhances recovery of muscle force-generating capacity following eccentric exercise. J. Sci. Med. Sport.

[19] Cooke M.B., Rybalka E., Stathis C.G., Cribb P.J., Hayes A. (2010). Whey protein isolate attenuates strength decline after eccentrically-induced muscle damage in healthy individuals. J. Int. Soc. Sports Nutr..

[20] Hansen M., Bangsbo J., Jensen J., Bibby B.M., Madsen K. (2015). Effect of whey protein hydrolysate on performance and recovery of top-class orienteering runners. Int. J. Sport Nutr. Exerc. Metab..

[21] Chen W.C., Huang W.C., Chiu C.C., Chang Y.K., Huang C.C. (2014). Whey protein improves exercise performance and biochemical profiles in trained mice. Med. Sci. Sports Exerc..

[22] Witard O.C., Jackman S.R., Kies A.K., Jeukendrup A.E., Tipton K.D. (2011). Effect of increased dietary protein on tolerance to intensified training. Med. Sci. Sports Exerc..

[23] Vegge G., Ronnestad B.R., Ellefsen S. (2012). Improved cycling performance with ingestion of hydrolyzed marine protein depends on performance level. J. Int. Soc. Sports Nutr..

[24] Phillips S.M. (2014). A brief review of higher dietary protein diets in weight loss: A focus on athletes. Sports Med..

[25] Rakobowchuk M., Tanguay S., Burgomaster K.A., Howarth K.R., Gibala M.J., MacDonald M.J. (2008). Sprint interval and traditional endurance training induce similar improvements in peripheral arterial stiffness and flow-mediated dilation in healthy humans. Am. J. Physiol..

[26] Sivasankaran S., Pollard-Quintner S., Sachdeva R., Pugeda J., Hoq S.M., Zarich S.W. (2006). The effect of a six-week program of yoga and meditation on brachial artery reactivity: Do psychosocial interventions affect vascular tone?. Clin. Cardiol..

[27] Okamoto T., Masuhara M., Ikuta K. (2007). Combined aerobic and resistance training and vascular function: Effect of aerobic exercise before and after resistance training. J. Appl. Physiol..

[28] Figueroa A., Park S.Y., Seo D.Y., Sanchez-Gonzalez M.A., Baek Y.H. (2011). Combined resistance and endurance exercise training improves arterial stiffness, blood pressure, and muscle strength in postmenopausal women. Menopause.

[29] Fekete A.A., Givens D.I., Lovegrove J.A. (2013). The impact of milk proteins and peptides on blood pressure and vascular function: A review of evidence from human intervention studies. Nutr. Res. Rev..

[30] Ballard K.D., Kupchak B.R., Volk B.M., Mah E., Shkreta A., Liptak C., Ptolemy A.S., Kellogg M.S., Bruno R.S., Seip R.L. (2013). Acute effects of ingestion of a novel whey-derived extract on vascular endothelial function in overweight, middle-aged men and women. Br. J. Nutr..

[31] Clifton P.M., Bastiaans K., Keogh J.B. (2009). High protein diets decrease total and abdominal fat and improve cvd risk profile in overweight and obese men and women with elevated triacylglycerol. Nutr. Metab. Cardiovasc. Dis. NMCD.

[32] Law M.R., Morris J.K., Wald N.J. (2009). Use of blood pressure lowering drugs in the prevention of cardiovascular disease: Meta-analysis of 147 randomised trials in the context of expectations from prospective epidemiological studies. BMJ.

[33] Weber T., Auer J., O’Rourke M.F., Kvas E., Lassnig E., Berent R., Eber B. (2004). Arterial stiffness, wave reflections, and the risk of coronary artery disease. Circulation.

[34] Miyachi M. (2013). Effects of resistance training on arterial stiffness: A meta-analysis. Br. J. Sports Med..

[35] Arciero P.J., Gentile C.L., Pressman R., Everett M., Ormsbee M.J., Martin J., Santamore J., Gorman L., Fehling P.C., Vukovich M.D. (2008). Moderate protein intake improves total and regional body composition and insulin sensitivity in overweight adults. Metabolism.

[36] Campbell W.W., Kim J.E., Amankwaah A.F., Gordon S.L., Weinheimer-Haus E.M. (2015). Higher total protein intake and change in total protein intake affect body composition but not metabolic syndrome indexes in middle-aged overweight and obese adults who perform resistance and aerobic exercise for 36 weeks. J. Nutr..

[37] Antonio J., Ellerbroek A., Silver T., Orris S., Scheiner M., Gonzalez A., Peacock C.A. (2015). A high protein diet (3.4 g/kg/day) combined with a heavy resistance training program improves body composition in healthy trained men and women—A follow-up investigation. J. Int. Soc. Sports Nutr..

[38] Antonio J., Peacock C.A., Ellerbroek A., Fromhoff B., Silver T. (2014). The effects of consuming a high protein diet (4.4 g/kg/day) on body composition in resistance-trained individuals. J. Int. Soc. Sports Nutr..

[39] MacKenzie-Shalders K.L., Byrne N.M., Slater G.J., King N.A. (2015). The effect of a whey protein supplement dose on satiety and food intake in resistance training athletes. Appetite.

[40] Gentile C.L., Ward E., Holst J.J., Astrup A., Ormsbee M.J., Connelly S., Arciero P.J. (2015). Resistant starch and protein intake enhances fat oxidation and feelings of fullness in lean and overweight/obese women. Nutr. J..

[41] Henderson G.C. (2014). Sexual dimorphism in the effects of exercise on metabolism of lipids to support resting metabolism. Front. Endocrinol..

[42] Rossi F.E., Fortaleza A.C., Neves L.M., Buonani C., Picolo M.R., Diniz T.A., Kalva-Filho C.A., Papoti M., Lira F.S., Freitas Junior I.F. (2015). Combined training (aerobic plus strength) potentiates a reduction in body fat but demonstrates no difference on the lipid profile in postmenopausal women when compared to aerobic training with a similar training load. J. Strength Cond. Res..

