# Supplementary material for: Protein-Pacing and Multi-Component Exercise Training Improves Physical Performance Outcomes in Exercise-Trained Women: The PRISE 3 Study
Source: Nutrients. 2016 Jun 1;8(6):332. doi: 10.3390/nu8060332 (PMC4924173; doi:10.3390/nu8060332)
Supplement: Supplementary file 1 [file nutrients-08-00332-s001.pdf]

Supplementary Materials: Protein-Pacing and Multi-Component Exercise Training Improves Physical Performance Outcomes in Exercise-Trained Women: The PRISE 3 Study

Paul J. Arciero, Stephen J. Ives, Chelsea Norton, Daniela Escudero, Olivia Minicucci, Gabe O'Brien, Maia Paul, Michael J. Ormsbee, Vincent Miller, Caitlin Sheridan and Feng He

Table S1. RISE Exercise Training Protocol.

Exercise	Type	Work	RPE	Monday	Tuesday	Wednesday	Thursday	Friday	Saturday /Sunday
**Resistance (R)**	WB	2–3 sets/exercise 10–15 reps	7–9	WB	---		---	---	
**Intervals (I)**	C	7–10 sets 30-60 s work 2–4 min rest	10/3	---	X	RECOVERY	---	---	RECOVERY
**Stretching (S)**	S	≤60 min	7–8	---	---		WB	---	
**Endurance (E)**	C	≥60min	6	---	---		---	X	

RPE: rating of perceived effort; C: choice of exercise modality; WB: whole body exercise; S: stretching exercise; X: exercise day. Exercise modalities available for C include: running, cycling, swimming, elliptical, rowing, cross-country skiing, *etc*.

Table S2. Sample Menus from the CON and PRISE nutritional intervention diet plans during the 12 week intervention. Menus were isocaloric and similar in meal timing.

	CON (1.0 g/kg·BW/Day)	PRISE (2.0 g/kg·BW/Day)
Breakfast	Steel cut oats, eggs; honey, nut/seed butter, fruit, caffeine beverage, One-A-Day Multivitamins®; 12 g protein; 30 g carbohydrate; 15 g fat	Eggs/egg whites, blueberries, coconut butter/milk, e+® caffeine beverage, Ageless Essentials®; 25 g protein; 15 g carbohydrate; 15 g fat
Mid-morning snack	Nature Valley Protein Chewy Bars®; 10 g protein, 14 g carbohydrate, 12 g fat	IsaPro®, 1.5 scoops, fresh fruit, 27 g protein; 3 g carbohydrate; 1.5 g fat
Lunch	Whole grain pita, tuna/turkey/chicken, baked chips, fresh fruit; 15 g protein; 30 g carbohydrate; 15 g fat	IsaLean Pro®: 36 g protein; 21 g carbohydrate; 6 g fat
Mid-Afternoon snack	Nature Valley Sweet and Salty Nut Granola Bars®, Horizon Organic Milk®; 12 g protein, 42 g carbohydrate, 10 g fat	IsaLean Bars®, ½ cup of yogurt or fruit; 18 g protein; 30 g carbohydrate; 5 g fat
Dinner	Fish/poultry/beef, whole grain rice/pasta or legumes, fresh vegetables, dried fruit, olive oil, water; 15 g protein; 30 g carbohydrate; 15 g fat	Fish/poultry/beef, fresh vegetables, chopped nuts, dried fruit, olive oil, milk; 25 g protein; 20 g carbohydrate; 15 g fat
Evening snack	Fresh fruit, nuts; 2-3 g protein; 20 g carbohydrate; 9 g fat	Greek yogurt, fruit; Ionix Supreme®; 5 g protein; 20 g carbohydrate; 5 g fat
Exercise Days *	Gatorade G2®, Electrolyte beverage	Replenish®, Electrolyte beverage

CON: protein based on 1.0g/kg·BW/day for a 64 kg·women; PRISE: protein based on 2.0g/kg·BW/day for a 64 kg·women. Meals were consumed ~3 h apart throughout the day.
